# Does surgeon sex and anthropometry matter for tool usability in traditional laparoscopic surgery? A systematic review and meta-analysis

**DOI:** 10.1007/s00464-023-10228-1

**Published:** 2023-07-11

**Authors:** Jaime Hislop, Dominic Orth, Oren Tirosh, Mats Isaksson, Chris Hensman, John McCormick

**Affiliations:** 1 Department of Mechanical Engineering and Product Design Engineering, Swinburne University of Technology, Melbourne, VIC Australia; 2 School of Health Sciences, Swinburne University of Technology, Melbourne, VIC Australia; 3 Institute for Health and Sport, Victoria University, Footscray, VIC Australia; 4 Department of Surgery, Monash University,, Melbourne, VIC Australia; 5 Department of Surgery, University of Adelaide, Adelaide, SA Australia; 6 Swinburne University of Technology, Melbourne, VIC Australia; 7 LapSurgery Australia, Melbourne, VIC Australia; 8 Centre for Transformative Media Technologies, Swinburne University of Technology, Melbourne, VIC Australia

**Keywords:** Gender, Discomfort, Ergonomics, Injury, Traditional laparoscopic surgery (TLS)

## Abstract

**Introduction:**

Hand size, strength, and stature all impact a surgeon’s ability to perform Traditional Laparoscopic Surgery (TLS) comfortably and effectively. This is due to limitations in instrument and operating room design. This article aims to review performance, pain, and tool usability data based on biological sex and anthropometry.

**Methods:**

PubMed, Embase, and Cochrane databases were searched in May 2023. Retrieved articles were screened based on whether a full-text, English article was available in which original results were stratified by biological sex or physical proportions. Article quality was discussed using the Mixed Methods Appraisal Tool (MMAT). Data were summarized in three main themes: task performance, physical discomfort, and tool usability and fit. Task completion times, pain prevalence, and grip style results between male and female surgeons formed three meta-analyses.

**Results:**

A total of 1354 articles were sourced, and 54 were deemed suitable for inclusion. The collated results showed that female participants, predominantly novices, took 2.6–30.1 s longer to perform standardized laparoscopic tasks. Female surgeons reported pain at double the frequency of their male colleagues. Female surgeons and those with a smaller glove size were consistently more likely to report difficulty and require modified (potentially suboptimal) grip techniques with standard laparoscopic tools.

**Conclusions:**

The pain and stress reported by female or small-handed surgeons when using laparoscopic tools demonstrates the need for currently available instrument handles, including robotic hand controls, to become more size-inclusive. However, this study is limited by reporting bias and inconsistencies; furthermore, most data was collected in a simulated environment. Additional research into how anthropometric tool design impacts the live operating performance of experienced female surgeons would further inform this area of investigation.

Tool usability considers how the physical characteristics, operating force, and intuitiveness of an instrument contributes to user comfort and performance [1]. This is impacted by the physical dimensions and strength of the user. Usability is defined as “the capability in human functional terms to be used easily and effectively by the specified range of users, given specified training and user support, to fulfill the specified range of tasks, within the specified range of environmental scenarios” [2]. Laparoscopic surgeons, like many professionals, are only as good as their tools. Their existing skill may be enhanced or limited by the operating equipment they use. Poor tool fit can cause pain or nerve injuries [3, 4]. Handle diameter relative to glove size can also impact the application of force with an instrument [5], making them more difficult to wield for those of above or below average hand size.

Even though pain and injury have been reported since the inception of TLS [3, 4], evolutions in handle design have occurred at a gradual pace. This is partly owing to the material and physical design constraints of surgical tools to guarantee sterilizability and ensure patient safety. Additionally, there is often more attention given to instrument functionality on the patient’s side than the surgeon’s side. Countless tool tips exist for tissue manipulation, dissection, cauterization, and morcellation. Comparatively few instrument manufacturers consider how effective tool use is impacted by the handle design, weight distribution, and balance of the tool, along with the physical dimensions of the user [6]. The elongated instruments required for TLS, the fulcrum effect created by the surgical ports, and an inadequate working height increase the shoulder abduction of those with a shorter arm span [7]. In their survey study, Morton et al. [8] reported that surgeons who were not between 160 and 184 cm tall and those who did not have a medium or large glove size were marginally more likely to experience pain and injury than those who did. These studies demonstrate that the well-documented ergonomic problems associated with TLS are exacerbated for surgeons of smaller stature and glove size.

Female surgeons are, on average, shorter, have a reduced arm span, wear a smaller glove size, and potentially have less strength than their male colleagues, creating a perfect storm of ergonomic challenges that could make them more susceptible to injury. Female medical students already choose nonsurgical specialties or leave training programs at a higher rate than males based on a desire for work-life balance, mentorship, a lack of female representation, or the experience of bias and discrimination [9, 10]. Ergonomic problems arising from regularly using tools designed for larger hands should not be another factor deterring women from pursuing lifelong surgical careers. As concerns exist regarding the sustainable provision of healthcare in the future [11, 12], it is vital to preserve the physical and mental health of surgeons by addressing a variety of factors including tool usability. This review investigates how the design of laparoscopic equipment impacts the comfort and performance of surgeons based on biological sex or anthropometry. The review collates results regarding task execution, surgeons’ pain, as well as tool design and usability stratified by biological sex, height, or glove size.

## Methods

This study does not require ethics approval from an Institutional Review Board (IRB) because it is a review based purely on the results of existing publications.

### Search strategy

A search of the PubMed, Embase, and Cochrane databases occurred in May 2023 using the following search strategy: (ergonomic OR ergonomics) AND (laparoscopic OR laparoscopy or minimally invasive) AND (anthropometric OR anthropometry OR gender OR women OR female OR glove size OR height) AND (surgeon OR physician OR surgeons OR trainees OR residents OR students). This review followed PRISMA guidelines [13]. Authors JH and DO independently screened the title and abstracts of articles for inclusion.

### Inclusion criteria

Studies were suitable for inclusion if they met the following criteria:

1. The full-text article was available in English;
2. The study contained original data;
3. Results regarding performance, discomfort, or tool usability during TLS were stratified based on biological sex, height, or glove size (although this was not required to be the primary outcome or purpose of the study).

The following exclusion criteria were used to screen studies:

1. Meta-analyses or review articles that only considered results of previous studies;
2. Studies that did not focus on TLS;
3. Studies that were only published as a supplemental abstract or summary;
4. Studies without a specific, numerical result regarding biological sex or anthropometry. Studies where similarity and differences were only included as a discussion, or a p-value was provided without accompanying numerical results were excluded (i.e., a statement such as “Women had significantly more symptoms than men (*p* < 0.01)” in isolation would not contain sufficient detail for inclusion).

If the assessments of the two authors were conflicting, the screening authors discussed their decisions until a consensus was reached.

### Reporting and methodological quality across studies

The Mixed Methods Appraisal Tool (MMAT) [14] was used by JH to consider study quality. The MMAT is not designed to provide a score for individual studies or be used to exclude studies that may be deemed to be of ‘low quality’. Rather, it is intended to identify potential points of concern regarding quality for multiple study designs. These designs include qualitative studies, quantitative studies that are randomized, non-randomized, or descriptive, and mixed methods studies.

### Data extraction and analysis

Study results were tabulated in Microsoft Excel by JH to identify trends in the outcome data across studies. Data only presented in a graphical form was approximated using WebPlotDigitizer [15]. Meta-analysis was performed for three main parts of the review. Firstly, several studies provided the completion times of standardized laparoscopic tasks stratified by biological sex. Forest plots of this information produced the mean difference in execution time across studies. Secondly, multiple studies reported the proportion of respondents experiencing discomfort while operating stratified by biological sex or height. The prevalence or odds ratios were collated into forest plots to determine existing trends in the risk of pain across studies. Thirdly, two studies reported on the grip styles used for the Harmonic Scalpel and LigaSure tools. Trends of one-handed, modified one-handed, and two-handed grips between male and female surgeons were assessed. For all three outcome types, meta-analysis was completed in OpenMeta[Analyst] using random effects models [16].

## Results

### Search results

**Fig. 1:**
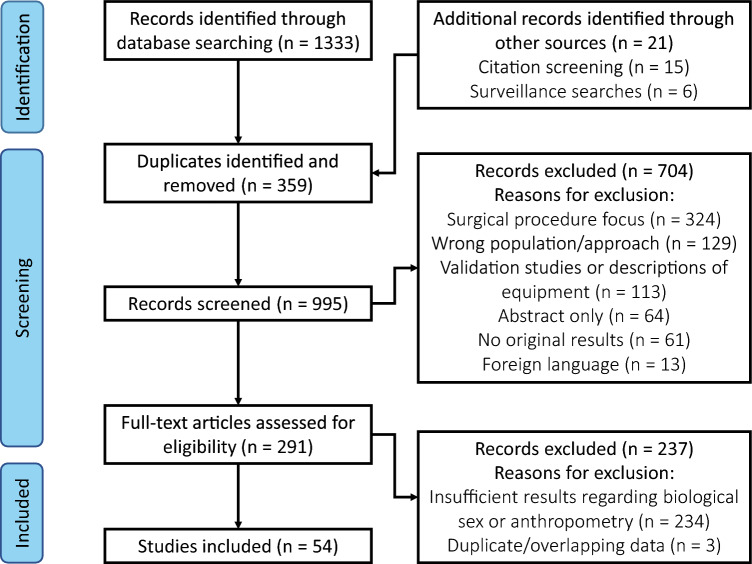
PRISMA diagram

In total, 1333 studies were identified from database searches. A further 21 studies were identified through reference screening and other sources. Three hundred and fifty-nine duplicates were removed. The abstracts of 995 studies were screened; 291 full-text articles were examined for inclusion. The full version was sought out for a high number of studies because comments on biological sex or anthropometry were often a secondary part of the analysis and not mentioned in the study abstract. A further 234 articles were excluded for not providing sufficient detail regarding differences based on biological sex or anthropometry (i.e. only a description or statistical significance without corresponding numeric results). Three studies were excluded for having duplicate or overlapping datasets as already included studies, despite being unique publications. Ultimately, 54 articles were included in total [17–70]. This process is depicted in Fig. 1.

### Study characteristics

The included articles contained a variety of data types, including surveys, task metrics, physical measurements and prototypes, and measures of muscle activity or strength. Sample size varied between three and 765. Differences based on biological sex or anthropometry was a primary focus in 35 out of 51 studies. Table 1 contains a summary of the included studies, organized based on the three main themes of this review: task performance, physical discomfort, and tool usability and fit.

**Table 1:** Characteristics of the included studies

Study	Year	*n*	Data type	Focus	Was biological sex/anthropometry a primary or secondary focus?
Task performance					
Bingener et al. [23]	2008	30	Task metrics	Difference in task performance between students with and without instruction on identifying common mistakes in laparoscopy	Secondary
Busshoff et al. [24]	2021	128	Task metrics	Difference in task metrics of male and female medical students and surgeons performing laparoscopic tasks with 2D and 3D visualization	Primary
Datta et al. [25]	2020	135	Task metrics	Difference in task metrics of medical students with and without gaming experience	Secondary
Donnon et al. [26]	2005	42	Task metrics	Difference in task metrics of medical students based on biological sex or the use of visualization exercises while developing laparoscopic skills	Primary
Elneel et al. [27]	2008	50	Task metrics	Difference in dexterity and ambidexterity of male and female medical students performing laparoscopic tasks	Primary
Flyckt et al. [28]	2017	51	Task metrics	Difference between predicted and actual FLS task scores depending on biological sex	Primary
Grantcharov et al. [29]	2003	25	Task metrics	Difference in task metrics of inexperienced residents performing laparoscopic tasks based on biological sex, gaming experience, and other factors	Primary
Hoops et al. [30]	2019	210	Task metrics	Difference in task metrics and autonomy of male and female residents and attendings	Primary
Kolozsvari et al. [31]	2011	32	Task metrics	Predictors of inherent laparoscopic skill	Primary
Madan et al. [32]	2008	51	Task metrics	Predictors of inherent laparoscopic skill	Primary
Mitchell et al. [33]	2019	64	Task metrics	Interest and aptitude for laparoscopy among medical students	Secondary
Nomura et al. [34]	2018	270	Task metrics	Predictors of inherent laparoscopic skill	Primary
Oussi et al. [35]	2021	47	Task metrics	Predictors of inherent laparoscopic skill	Primary
Shane et al. [36]	2008	26	Trials to proficiency	Difference in performance and skill acquisition between gamers and non-gamers	Secondary
Strandbygaard et al. [37]	2013	99	Task metrics	Difference in performance and skill acquisition depending on whether participants received instructor feedback	Secondary
Thorson et al. [38]	2011	32	Task metrics	Difference in task metrics of male and female medical students	Primary
Van Hove et al. [39]	2008	35	Task metrics	Predictors of inherent laparoscopic skill	Primary
White and Welch [40]	2012	132	Task metrics	Difference in performance and skill acquisition of male and female medical students and residents performing laparoscopic tasks	Primary
Physical discomfort					
Adams et al. [41]	2013	495	Survey results	Pain prevalence and impact	Secondary
AlSabah et al. [42]	2019	113	Survey results	Pain prevalence and impact	Secondary
Dalager et al. [43]	2019	284	Survey results	Pain prevalence and impact	Secondary
Franasiak et al. [44]	2012	260	Survey results	Pain prevalence and impact	Secondary
Galindo et al. [45]	2021	140	Survey results	Pain prevalence and impact	Primary
Gutierrez-Diez et al. [46]	2018	129	Grip strength and survey results	Pain prevalence and impact	Secondary
Hignett et al. [47]	2017	Part 1: 67 Part 2: 11	Part 1: survey results Part 2: observation and interviews	Pain prevalence and postural assessment	Primary
Janki et al. [48]	2017	127	Survey results	Pain prevalence and impact	Secondary
Kapoor et al. [49]	2021	765	Survey results	Pain prevalence and impact	Primary
Lloyd et al. [50]	2019	701	Survey results	Pain prevalence and impact	Secondary
McDonald et al. [51]	2014	350	Survey results	Pain prevalence and impact	Secondary
Quinn and Moohan [52]		53	Survey results	Pain prevalence and impact	Secondary
Shepherd et al. [53]	2016	50	Survey results	Pain prevalence and impact	Secondary
Sutton et al. [54]	2014	314	Survey results	Pain prevalence and impact	Primary
Wong et al. [55]	2022	190	Survey results	Pain prevalence and perception of instrument fit based on biological sex	Primary
Zehetner et al. [56]	2006	8	Physical measurements and joint angles	Angle of cervical spine while using monitor at standard and preferred height	Primary
Tool usability and fit					
Adams et al. [57]	2008	65	Survey results	Laparoscopic instrument ease of use according to biological sex and glove size	Primary
Armijo et al. [58]	2022	18	Electromyography (EMG)	Difference in muscle activity between experienced male and female laparoscopic surgeons	Primary
Berguer and Hreljac [59]	2004	726	Survey results	Laparoscopic instrument ease of use according to glove size	Primary
DiMartino et al. [60]	2004	22	Physical measurements	Optimal handle diameter and configuration based on hand size	Primary
Du et al. [61]	2023	Part 1: 21 Part 2: 30	Hand measurements, prototype design, EMG, motion capture, and subjective rating	Prototype tool design and evaluation based on 3D scanned hand anthropometry	Primary
Filisetti et al. [62]	2015	138	Survey results	Laparoscopic instrument ease of use according to glove size	Primary
Gonzalez et al. [63]	2015	135	Ranking following simulated task	Difference in preferred handle size based on hand/glove size	Primary
Green et al. [64]	2022	58	Hand measurements and survey results	Perception of instrument discomfort and difficulty based on hand anthropometry	Primary
Kasai et al. [65]	2013	11	Grip strength and EMG	To assess and ultimately improve laparoscopic grasper design	Primary
Kono et al. [66]	2012	241	Survey results	Laparoscopic instrument ease of use according to biological sex and glove size	Primary
Kono et al. [67]	2014	113	Grip strength and stapler activation force	Compare hand length and grip force with stapler activation force	Primary
Kono et al. [68]	2022	3	Prototype design	Customization of handle design for female surgeons	Primary
Matern and Waller [69]	1999	10	Existing anthropometric data, force measurements, grip characteristics, prototype design	Consideration of design of commercially available tool handles and prototype based on anthropometric data	Primary
Ordóñez-Ríos et al. [70]	2019	7	Physical measurements	Ergonomics of operating theater based on surgeon's anthropometry	Primary
Sreekanth et al. [71]	2019	120	Physical measurements	Prototype tool design based on hand anthropometry	Primary
Sreekanth et al. [72]	2020	282	Prototype design and subjective rating	Prototype tool design and feedback for customized laparoscopic handle	Primary
Stellon et al. [73]	2017	58	Hand measurements	Consideration of design of commercially available tool handles based on participants' anthropometric data	Primary
Sun et al. [74]	2014	14	Hand measurements, prototype design, EMG, motion capture, and subjective rating	Comparison of prototype to existing tool handle using subjective and objective measures	Primary
Weinreiche et al. [75]	2022	488	Survey results	Perception of instrument fit based on glove size	Primary
Wong et al. [76]	2022	38	Grip strength and survey results	Changes in applied force, workload and discomfort while using bipolar surgical tools	Primary

### Study quality

#### Quantitative descriptive studies

This review included 20 surveys, classified as quantitative descriptive studies [35–40, 42–49, 51, 53, 56, 58, 60, 69]. Areas of concern highlighted by the MMAT are sampling strategy, representation, appropriateness of measurement and analysis methods, and non-response bias. No survey studies reported performing a sample size calculation prior to commencement, challenging the statistical strength of these studies. Sample frames for the survey studies included surgical societies [35, 38, 42–45, 48, 49, 53, 56, 60], social media [36, 69], or local universities and hospitals [37, 39, 40, 46, 47, 49, 51, 58]. Five studies considered representation and compared the demographics of their respondents in relation to the rest of the target population [38, 43, 46, 49, 60]. Surveys, when provided, were considered relevant for addressing the research aims. Some surveys were adapted from the previously validated Nordic Musculoskeletal Questionnaire (NMQ) [35–37, 39, 40] or existing unvalidated surveys [39, 45, 47, 69]. The remaining studies used original surveys [38, 42–44, 46, 48, 49, 51, 53, 56, 58, 60]. Survey validation with a smaller cohort was only mentioned in three articles [35, 37, 43]. Results were generally given as a prevalence, mean and standard deviation, or median and interquartile range. These types of tabulated data were easily understood and appropriate. Most studies provided a full [38–40, 45–47, 49, 51, 58] or partial [35, 42, 48, 53, 60, 69] set of p-values to demonstrate significant differences based on demographic factors or the presence of symptoms. Eight studies performed univariate and multivariate analysis to consider how factors such as biological sex or glove size contributed to risk of injury [35, 37, 38, 43, 45, 46, 48, 49]. Stated response rates varied between 0.62 [69] and 64.2% [40]. Two studies based this calculation on the number of opened emails rather than sent emails, increasing their response rate [35, 44]. Six studies recognized the possibility of nonresponse bias [37, 38, 42–44, 53], although only three provided a calculation for how this may impact results [37, 38, 43].

#### Quantitative randomized controlled trials

Four of the included studies were randomized control trials [17, 18, 20, 31]; in these articles results relating to biological sex or anthropometry were secondary outcomes. The MMAT focuses on the randomization process, initial comparability, blinding, compliance, and completeness of results. Regarding randomization, one study reported using the permuted block technique [17]; other studies made more general comments about using an independent researcher [18] or computer randomization [31]. Following randomization, resulting groups were balanced regarding biological sex in three articles [17, 20, 31]. The remaining study was a crossover trial meaning all subjects participated in both experimental conditions. Two studies reported similar baseline performance between groups [17, 20]. Only Strandbygaard et al. [31] mentioned concealing the groupings between allocation and data collection, although others stated their methods of blinding the investigators to the experimental condition of the participants [17, 18]. Regarding compliance, Busshoff et al. [18] excluded results from some tasks that were “not done according to study protocol”. Donnon et al. [20] required participants to document their adherence to the intervention of practising visualization exercises in the week between data collection sessions; however, compliance was not reported in the study results. Strandbygaard et al. [31] reported dropout before and during trials. Otherwise, it was assumed authors were reporting complete outcome data.

#### Quantitative non-randomized studies

Twenty-nine non-randomized studies were included in this review [19, 21–30, 32–34, 50, 52, 54, 55, 57, 59, 61–68, 70]. For this study design, the MMAT considers appropriateness of measurement and analysis methods, administration of or exposure to intervention, completeness of results, accounting for confounders, and generalizability. Data collection methods included physical measurements [50, 55, 59, 61, 63–67], EMG [52, 55, 59, 68], dynamometry [59, 61, 63, 70], and performance metrics [19, 21–30, 32–34, 55]. All these measurement methods appeared to be relevant and appropriately controlled. Seven studies utilized questionnaires to refine tool handle prototypes [54, 55, 57, 62, 65, 66, 68]; by nature these studies contained more subjectivity. There was no concern about adherence to an experimental condition because in most studies participants were grouped based on inherent qualities, rather than an applied intervention. The factors of interest (including biological sex, physical proportions or strength, handedness, and surgical experience) did not change during the cross-sectional study [19, 21–30, 32–34, 50, 52, 54, 55, 57, 59, 61–68, 70]. Regarding completeness, a few studies reported dropout [24, 33] or incomplete datasets [19, 21]. Studies explicitly referred to minimizing the impact of confounding variables by standardizing the experimental setup and protocol [21, 61], including results from larger anthropometric studies [63, 67], controlling for demographic factors between participant groups [24, 52], or using statistical analysis to explore demographic factors that potentially influenced results [19, 22, 23, 25, 27, 28, 32, 33, 57]. Only five studies compared the demographics, proportions, or performance of their participants to other cohorts or the wider population to discuss generalizability [24, 27, 61, 64, 67].

#### Mixed methods studies

The study by Hignett et al. [41] was the only one that used a mixed methods approach, utilizing surveys, postural assessment, and semi-structured interviews. The survey was based on previous studies [38, 45]. A brief description of the results was provided without statistical analysis. Generalizability, sample size, and nonresponse bias were not discussed. A validated postural scoring tool was used to assess participants with a broad range of statures, and results were presented in full. Confounding variables may have been created by the difficulty of simulating positioning while operating. Descriptive interviews exploring theater and patient factors were conducted followed by NVivo thematic analysis [71], although insufficient detail regarding the interview structure and thematic analysis results were provided.

### Meta-analysis findings

#### Task performance

**Fig. 2:**
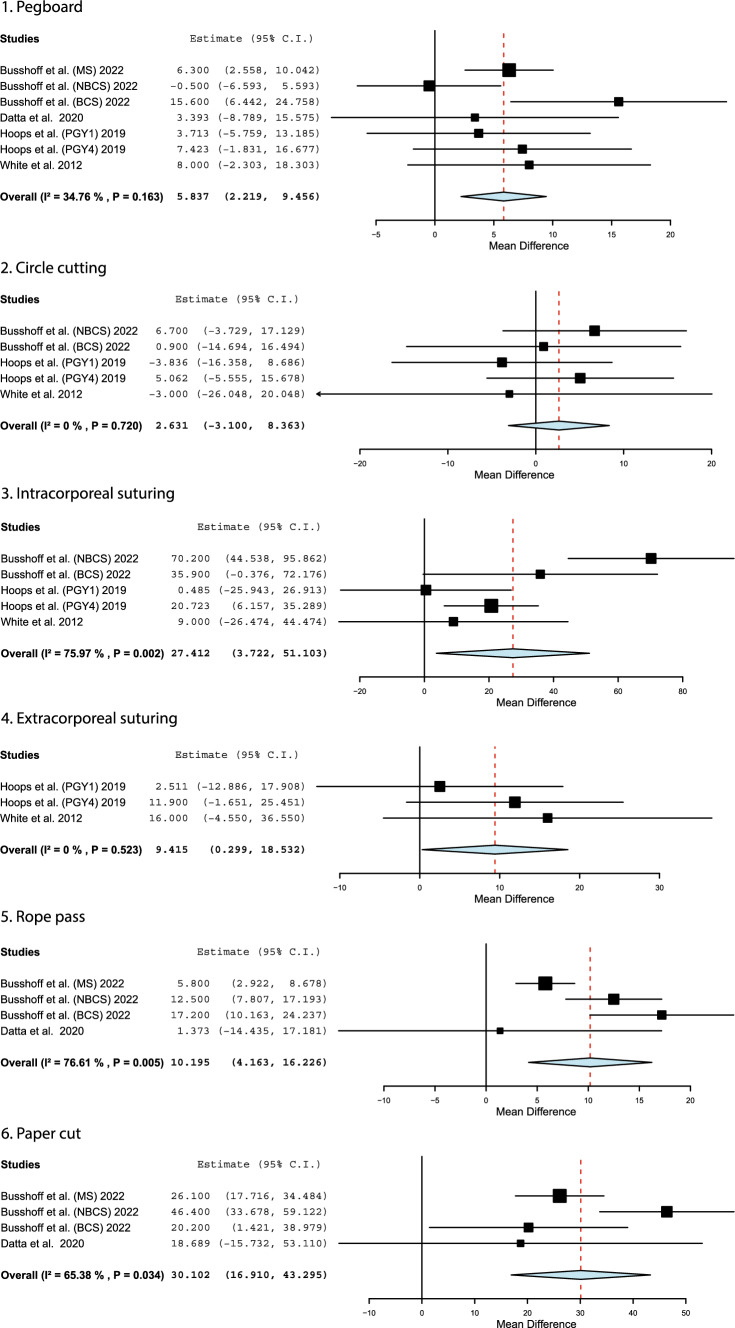
A meta-analysis depicting the mean difference of task completion times, measured in seconds, between male and female participants [18, 19, 24, 34]. Results from two studies are separated by Postgraduate Year (PGY), Medical Students (MS), Non-Board-Certified (NBCS), and Board-Certified Surgeons (BCS). A positive mean difference indicates female surgeons took longer to finish simulated exercises

Eighteen studies considered the difference in completion times and other task metrics between male and female participants. Experience levels included novices or medical students [17–21, 25–30, 32, 34], surgical residents [22–24, 33, 34], and practicing surgeons [18]. Several studies presented completion times for standardized laparoscopic parkour [72] or Fundamentals of Laparoscopic Surgery (FLS) [73] tasks and were combined in a meta-analysis. The collated results from Busshoff et al. [18], Datta et al. [19], Hoops et al. [24], and White and Welch [34] are presented in Fig. 2. These forest plots show that female surgeons took significantly longer to complete the pegboard, suturing, rope pass, and papercut tasks than their male colleagues. The mean difference in task completion times in seconds ranged from 2.63 to 30.1 s.

In addition to the meta-analysis results, multiple other studies reported increased completion times and path lengths for female participants. Bingener et al. [17] had novices complete six repetitions of a suturing exercise, and the female subjects consistently took an average of 25 s longer than the male subjects to complete the exercise across groups and repetitions. Donnon et al. [20] required individuals to complete dextrous suturing and bead maneuvring tasks under different visualization conditions. In the first session, the mean difference between sexes was 8–44 s; the female cohort was consistently slower than their male counterparts. After a 1 week break the disparity increased, ranging from 50 to 133 s. The median results from Elneel et al. [21] and Grantcharov et al. [23] suggested that male participants were 11–27% faster with fewer unnecessary movements during tasks. The number and severity of errors was comparable between sexes for both studies. Nomura et al. [28] had a similar finding, with female medical students taking 9.6 s longer to complete the pick-and-place task (*p* = 0.0565) and moving their instruments 257–367 mm further (*p* < 0.02). Mitchell et al. [27] found that females took 3.5 s longer to complete the peg transfer task, although this difference was not significant. Shane et al. [30] investigated trials to proficiency in pick-and-place and object passing tasks on the Minimally Invasive Surgical Trainer (MIST) system. Across tasks, females took a median of 10 more trials than males to demonstrate proficiency (*p* = 0.006). Thorson et al. [32] had 32 participants (16 male and 16 female) perform six repetitions of an object passing task at two different difficulty levels. Median MIST scores differed by 11–40 points, with female participants performing significantly worse on all metrics measured by the trainer. Madan et al. [26] compared the box trainer and MIST-VR performance of participants based on various hobbies potentially impacting dexterity, such as sewing or gaming, as well as biological sex. Results showed that practicing dextrous activities improved MIST-VR scores, although not significantly. Male participants achieved significantly higher MIST-VR scores than females.

In contrast, neither Oussi et al. [29], Kolozsvari et al. [25], nor Flyckt et al. [22] found a significant difference in task performance based on biological sex. Oussi et al. [29] reported that although females consistently received higher scores across three simulated diathermy tasks than males, this disparity was not significant and decreased with each attempt. Kolozsvari et al. [25] examined the learning curve of medical students and found that biological sex had no difference on the initial and final peg transfer task scores, or the rate of skill acquisition. Flyckt et al. [22] examined confidence between male and female surgical residents by having them complete FLS tasks and comparing the difference between their predicted and actual scores. Actual tasks scores were statistically similar between sexes. Despite performing equally well, the initial predictions of female residents had underestimated their performance by 11.1–22.5 points, whereas males had overestimated their scores by 4.2 points on average.

Long-term or follow-up data was available for at least some of the participants involved in the studies by Hoops et al. [24], Strandbygaard et al. [31], and Van Hove et al. [33] over varying time periods. Hoops et al. [24] measured FLS task performance annually between the first and fourth postgraduate years of their students. Dropout was observed over this time; there were only 23 participants at the final timepoint from the original 107. The results by Hoops et al. suggest that the gender disparity increased between the first and fourth postgraduate years, as shown in Fig. 2. Strandbygaard et al. [31] considered task performance based on whether students received feedback or not. Initial results showed that a longer time was required for female trainees to reach proficiency; this was only significant between the groups that did not receive feedback. The 6-month follow-up data, published in another study that otherwise did not meet the inclusion criteria for this review, showed no significant differences between biological sexes [74]. Van Hove et al. [33] reported the McGill Inanimate System for Training and Evaluation of Laparoscopic Skills (MISTELS) score pre and post-training, as well as one year later. Similar performance was observed between biological sexes during the training period; however, the female surgeons performed significantly better at follow-up. More specifically, the average MISTELS score increased by 31 points for the female cohort, whereas it decreased by 46 among the male cohort.

#### Physical discomfort

The percentage of female respondents within survey studies ranged from 5.3 [36] to 78.4% [49]. Across studies, the height, glove size, and experience were all generally lower for female surgeons compared to their male colleagues. The likelihood of experiencing physical symptoms due to biological sex or anthropometry was presented within articles in the form of prevalence or odds ratios. The relative risk of discomfort between biological sexes varied significantly between studies. Sutton et al. [48] reported that males were twice as likely to experience pain in the lower extremities, whereas Wong et al. [49] found that females were five times more likely to experience pain overall. After accounting for additional factors including glove size, height, age, experience, caseload, and case length, the relative risk of discomfort between biological sexes ranged from statistically insignificant [35, 48, 49] to a sevenfold increase in the risk of female surgeons experiencing physical symptoms [38] across studies.

**Fig. 3:**
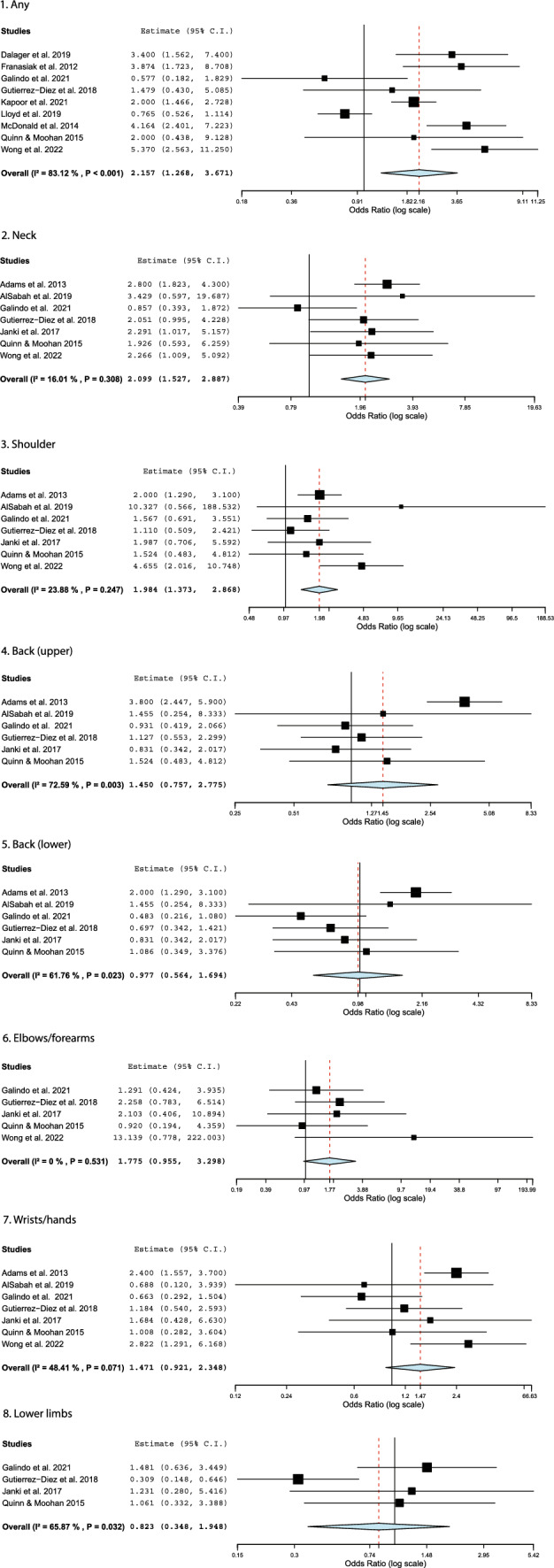
A meta-analysis of pain prevalence by anatomic region and biological sex [35–40, 42, 44–46, 49]. An estimated odds ratio greater than one indicates a larger proportion of female surgeons reporting pain

Prevalence estimates for pain ranged from 39 [44] to 92.5% [38] for female surgeons and 46% [44] to 89% [40] for male surgeons. In most instances the proportion of females reporting symptoms was larger than males. Prevalence and odds ratio data were combined in a meta-analysis to examine trends across studies [35–40, 42, 44–46, 49]. Figure 3 depicts the risk of female and male surgeons experiencing symptoms stratified by anatomic region. Overall, the odds ratio of female laparoscopists to experience injury was 2.16(1.27, 3.67) across studies. More specifically, female surgeons were twice as likely to report neck and shoulder pain than their male colleagues, a statistically significant difference. Male and female surgeons were equally likely to experience lower back pain. The lower limbs were the only site where males were more likely to experience discomfort; however, this difference was not significant.

Shepherd et al. [47] found that female surgeons experienced significantly more discomfort while operating than their male colleagues. This trend existed regardless of case length although the disparity increased during longer procedures. Those with a smaller glove size also reported more pain, but the difference was only significant during shorter operations.

**Fig. 4:**
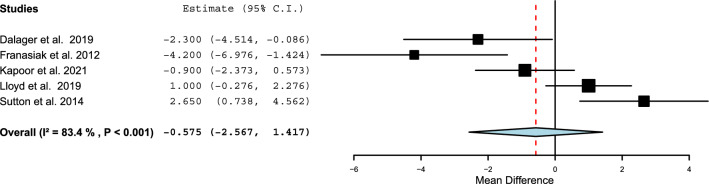
A meta-analysis of the average height in centimeters of surgeons reporting pain [37, 38, 43, 44, 48]. A positive mean difference indicates that surgeons experiencing symptoms were taller on average

Several survey studies presented the mean height of those experiencing physical discomfort compared to those without pain. Sutton et al. [48] and Lloyd et al. [44] reported that surgeons with symptoms were taller on average, whereas Kapoor et al. [43], Franasiak et al. [38], and Dalager et al. [37] found the opposite result. There was no observable trend across studies. Figure 4 depicts this information. Hignett et al. [41] and Zehetner et al. [50] examined the impact of height on surgeons’ posture using physical measurements and observation. Hignett et al. [41] scored the posture of 11 surgeons between 158.8 and 189.4 cm tall using a Rapid Entire Body Assessment (REBA) with three different surgical port configurations and two different abdomen depths representing 50^th^ and 99^th^ percentile Body Mass Indexes (BMIs). REBA scores ranged between one and four, which are acceptably low-risk. By performing an additional Pearson correlation test on the results provided by Hignett et al., shorter surgeons obtained significantly higher REBA scores for the 50^th^ percentile BMI abdominal cavity with the midline and bilateral port placements, configurations that would possibly require the surgeon to rotate their back or bend over the patient. Zehetner et al. [50] used the measurements of eight participants’ eye level as well as preferred and maximum monitor heights to extrapolate possible neck angles of operating surgeons. Raising the monitor by 1 cm would increase the neck angle by 0.48° when positioned 120 cm from the monitor. At the preferred monitor height, calculated neck angles were 4.8°–8.5° below the horizontal plane. At the maximum monitor height, neck angles were 2.9°–14° above the horizontal plane depending on the surgeon’s stature.

Overall the included survey studies demonstrate that female surgeons are at a higher risk of injury. No trend was found regarding surgeon height and discomfort from questionnaire data, although Hignett et al. [41] and Zehetner et al. [50] demonstrated how shorter surgeons may be adversely impacted by trocar position and monitor height. Contributing factors for injury may include poor tool fit and the need to apply excessive force to properly operate instruments, which were investigated in the studies summarized below.

#### Tool usability and fit

**Fig. 5:**
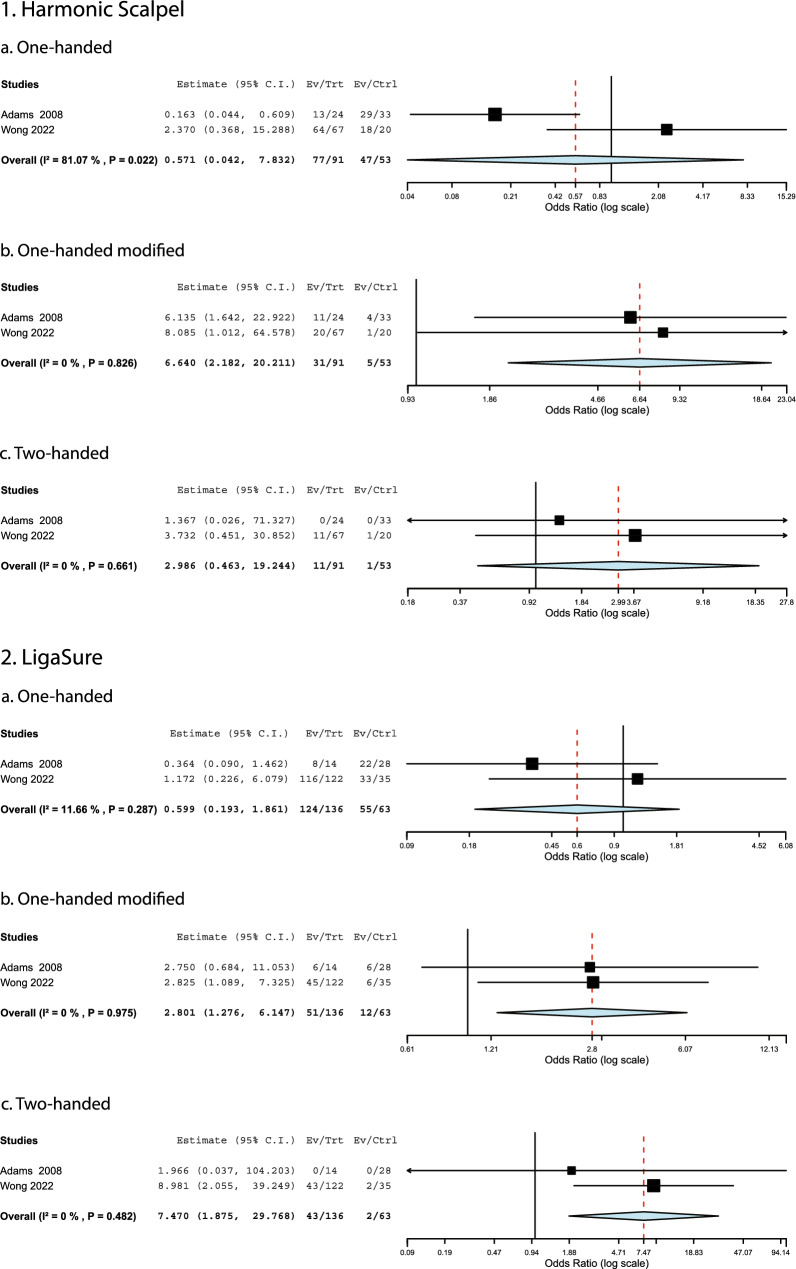
A meta-analysis of grip style for the Harmonic Scalpel and LigaSure instruments [49, 51]. An odds ratio above one indicates females were more likely than males to use the indicated grip style

Adams et al. [51], Berguer and Hreljac [53], Filisetti et al. [56], Green et al. [58], Weinreiche et al. [69], and Wong et al. [49] examined self-reported tool usability and grip styles. Figure 5 depicts a meta-analysis of grip style results from Adams et al. [51] and Wong et al. [49] for the Harmonic scalpel and LigaSure tools. Female surgeons were 2.8–7.5 times more likely to use a modified one-handed or two-handed grip than their colleagues. Similar trends were reported for other instruments [49, 51]. Additionally, female surgeons reported that laparoscopic tools were too large and awkward to use more frequently than male surgeons [49, 51]. Regarding glove size, which is correlated with gender, Berguer and Hreljac [53] and Kono et al. [60] reported a 4.3–28.8% increase in the proportion of time that surgeons who wore size 6.5 or smaller reported difficulty when using different instruments compared to those with a larger glove size. Filisetti et al. [56] found that surgeons with a glove size between 7 and 8 consistently provided lower difficulty scores than those with smaller or larger glove sizes. The retrieval bag had the highest difficulty score for all glove sizes. Green et al. [58] found that all female respondents reported difficulty using surgical tools, compared with only 56% of males (*p* < 0.001). Significant correlations were found between increased difficulty, pressure, and fatigue and shorter finger measurements (not including the thumb). Weinreiche et al. [69] showed that 53% of those with a glove size of 6.5 or below reported difficulty with laparoscopic tools, compared to 32% of those with larger hands. Multivariate analysis showed that female surgeons or those with a glove size less than 7.0 were 3 to 5.5 times more likely to report difficulty with surgical tools. Being a female surgeon or having a smaller glove size were consistently associated with increased difficulty using laparoscopic tools across studies.

Armijo et al. [52] and Kasai et al. [59] both utilized Electromyography (EMG) in their studies. Commonly, EMG data is used in ergonomic investigations to examine muscle activity and force. It is normalized and presented as a percentage of the Maximum Voluntary Contraction (%MVC) obtained during a controlled exercise. Additionally, EMG data may be analyzed in the frequency domain, with a reduction in the median frequency over time indicating muscle fatigue. Kasai et al. [59] showed that female surgeons use 100–122%MVC in their forearm muscles when operating a laparoscopic stapler. Armijo et al. [52] obtained %MVC data to examine muscle activity, median frequencies to examine muscle fatigue, and survey data. The muscle activity of female participants was significantly higher for the upper trapezius, flexor carpi radialis, and extensor digitorum muscles, indicating increased strain. The median frequencies of the EMG signal were significantly higher for the upper trapezius for the female cohort and the extensor digitorum for the male cohort. However, no significant change in median frequency was observed over time, suggesting the participants did not experience muscle fatigue. The perceived fatigue of female surgeons was significantly higher on several facets of the questionnaire.

Matern and Waller [63], Gutierrez-Diez et al. [40], Kasai et al. [59], Kono et al. [61], DiMartino et al. [54], Gonzalez et al. [57, 75], and Wong et al. [70] examined grip strength and diameter. Matern and Waller [63] investigated pinch grip between the thumb and each other finger on both hands of participants. The strongest grip was achieved between the thumb and middle fingers bilaterally. Male participants had a 3–73% stronger pinch grip than female participants. Across studies, females could only apply 62–67% of the force applied by their male counterparts on a dynamometer when using a power grip [40, 61, 70]. The study by Kasai et al. [59] only included female participants, who had a maximum strength of 266 ± 67 N when gripping the dynamometer. However, when using a laparoscopic stapler, participants could only exert 148 ± 40 N because the handle diameter before compression (11 cm) was twice the size of the dynamometer (5.3 cm). Kono et al. [61] found that the ideal grip diameter, based on the cylinder width at which the greatest amount of force could be applied to a dynamometer, was 6.25–6.35 cm for males and 5.41–5.55 cm for females. DiMartino et al. [54] and Gonzalez et al. [57] both had participants elect their preferred handle diameter by having them grip or use prototypes of varying widths. This subjective assessment resulted in an ideal width between 2.9 and 5.7 cm. When comparing the optimum handle diameter with the participants’ anthropometric data in an additional publication, Gonzalez et al. found a consistent ratio between palm length and grip width of 2.97:1 [75]. Wong et al. [70] reported that the grip strength of all participants decreased to approximately 80% over a two-minute period using various bipolar surgical tools. Significantly greater decreases in strength as well as increases in workload and discomfort were found consistently for participants with a glove size less than 7 compared to those with larger hands.

**Fig. 6:**
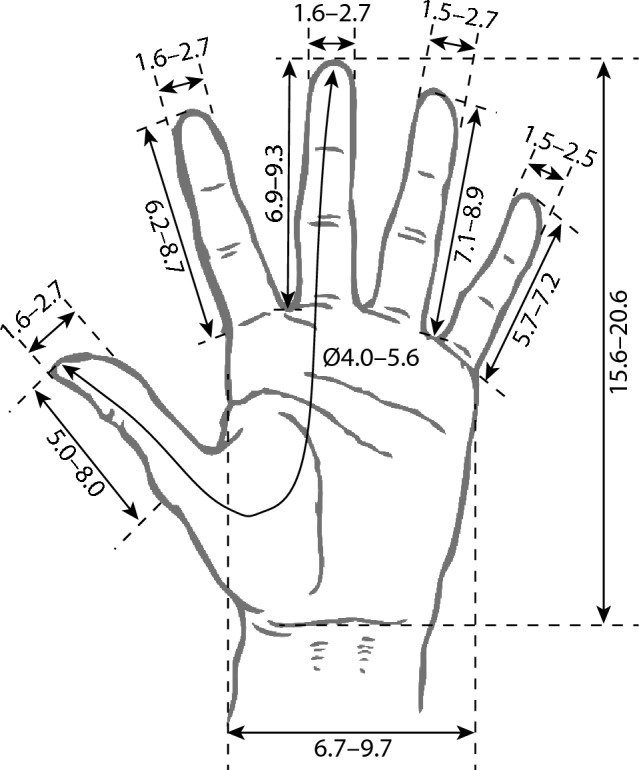
The 5th and 95th percentiles of hand anthropometry presented across studies in centimeters [55, 58, 59, 63–65, 67]

Figure 6 shows the most extreme values for the 5th and 95th percentiles of hand measurement based on data from Du et al. [55], Green et al. [58], Kasai et al. [59], Kono et al. [61], Matern and Waller [63], Ordóñez-Ríos et al. [64], Sreekanth et al. [65], and Stellon et al. [67]. Some of these values were taken from the surgeons participating in the included studies, other measurements came from larger anthropometric investigations referenced within the articles. Hand width and length varied by 3 cm and 5 cm, respectively. Finger width and length varied by 1.1 and 3 cm, respectively. Stellon et al. [67] was the only study to provide results regarding grip diameter, which ranged from 4 to 5.6 cm. These measurements would have a bearing on the comfort and usability of laparoscopic tools.

Du et al. [55], Kono et al. [62], Matern and Waller [63], Sreekanth et al. [65, 66], and Sun et al. [68] presented handle prototypes based on anthropometry and ergonomic considerations. These authors had different design priorities, as summarized in Table 2. Some overarching themes that existed across studies were to reduce the weight and size of the tool, allow adjustments or customization to fit the user’s hand size, and restore a neutral wrist position. Only Du et al. [55], Sreekanth et al. [66], and Sun et al. [68] presented results regarding the difference between their prototype and existing tool handles. Du et al. [55] demonstrated that their refined prototype performed equally well regarding task time and performance, while reducing the muscle load on the trapezius and increasing satisfaction compared to a similar commercial product. Sreekanth et al. [66] administered a five-point Likert scale survey to 80 participants and found an average increase in perceived grip, functionality, comfort and wrist posture of 1.5–2.3 points. It should be noted that only male surgeons participated in this study. Sun et al. [68] evaluated EMG, motion capture and survey data from eight participants. Minor, inconclusive changes in muscle activity, wrist angle, and subjective ratings were found.

**Table 2:** Summary of tool prototypes

Prototype	Design goals/modifications
Du et al. [61]	Optimize application of force by utilizing pistol grip and considering thumb position
	Reduce pressure areas
	Consider finger range of motion to reduce overlap and improve flexibility
	Reflect natural hand grip and minimize stretching by considering distance between the index finger and thumb
Kono et al. [68]	Reduce the weight and dimensions of purse string suture instrument to accommodate female surgeons
	The prototype had a 26.4% reduction in weight and 15 mm reduction in length compared to the existing tool
Matern and Waller [69] (1) (2)	Create an intuitive, multifunctional, and adjustable tool handle
	Minimize and optimize dimensions to facilitate one-handed operation
	Position controls so they are accessible and operated by the most sensitive areas of the hand
	Ensure instrument shaft is in line with the forearm’s longitudinal axis
Sreekanth et al. [71, 72]	Add palm support to increase handle contact area and grip force
	Reorient thumb loop to improve thumb and wrist position
	Use rubber inserts to accommodate different hand sizes
	Reduce tool mass (not achieved)
Sun et al. [74]	Reorient handle direction to straighten wrist position
	Increase finger loop size to accommodate middle and ring fingers
	Bevel edge of thumb loop to accommodate angled position of thumb

## Discussion

This review examined the design of laparoscopic tools in terms of performance, comfort, and usability. The first meta-analysis results showed that females completed all simulated tasks significantly slower than their male counterparts except for circle cutting. Possible reasons for longer completion times could be the need for female participants to adjust their grip while operating, the increased time or exertion required for them to successfully activate laparoscopic tools, or the learning curve when considering novices. It should be noted that most of the included studies only recruited medical students and surgical residents, so the impact on experienced surgeons during surgical procedures is unclear. While a difference of 30 s may not be noteworthy, in the context of a 5 min task this represents a 10% increase in the completion times of female participants. It could be speculated that there may be a cumulative effect during procedures leading to longer operative times [76]. Female surgeons are unquestionably skilled; their surgical outcomes and complication rates are the same as their counterparts [77, 78]. However, it is still vital to consider how they are impacted by the tools they use to operate.

The effect of using 3D visualization and robotics on surgical skill acquisition has been explored in the literature. Busshoff et al. [18] examined the impact of 3D visualization on the task completion times of medical students and surgeons and found that females’ times improved by 27.8% compared to 25.3% for males (*p* = 0.005), although males were still faster overall. Chiu et al. [79] showed that female medical students achieved significantly better results on a da Vinci simulator during a suture sponge exercise on all metrics except needle drops, achieving scores 50 points lower than their colleagues. These studies provide limited support for the use of surgical robotics to improve design equity in the operating theater.

Female surgeons experienced neck and shoulder pain significantly more frequently than their male colleagues. This may be related to increased neck flexion for shorter surgeons [50] and shoulder abduction for those with a smaller arm span [7] or physically supporting and operating the laparoscopic tools. The peak operating force of laparoscopic staplers, reported as requiring 250 N [59] or 21.8–42.3 kg [61], may place increased strain on female and small-handed surgeons. Even if female surgeons can surpass these thresholds in some circumstances, force is inextricably linked to grip diameter [59]. If female surgeons are required to operate with tools they find large and awkward to use [49, 51], this will place an artificial limitation on their maximum producible force. It may be impossible to accommodate everyone when considering tool dimensions, given that hand length and width vary by several centimeters between the 5th and 95th percentiles of measurements; however, efforts should be increased to create adjustable, body-scalable instruments that are suitable for 95% of the surgical population [80].

This review highlights the inadequacy of tool design and fit for many surgeons. Studies used survey data, physical measurements, EMG, and grip force to demonstrate the shortcomings of existing instrument handles for female and small-handed surgeons. Several studies sought to address this by creating new prototypes based on physical measurements. Multiple options for collecting anthropometric data were used across studies, varying in complexity. Sreekanth et al. [66] reported 95% accuracy by simply tracing the hands of participants with pen and paper. Such a fast method requiring little skill and no specialist equipment opens up possibilities for increasing communication between surgeons and manufacturers to improve tool fit. A step beyond this would be utilizing the scanning and scaling technology available in smartphones which is already being used to customize clothing and other products [81] to measure the dimensions of surgeons’ hands remotely. Direct measurement methods with tape measures, callipers, or scanning devices are also possible, although they require additional tools, time, and the physical presence of the surgeon. Lam and Huang [82] presented the possibility of taking measurements from plaster casts of the hand in certain grip positions. This method would be difficult to implement on a large scale, although may provide more dynamic information about the hand. If rapid and customizable prototyping techniques could be utilized in the strict regulatory environment of the operating theater, the comfort and efficiency of surgeons performing TLS would significantly improve.

This study has several limitations. Firstly, most existing studies assessing differences in performance based on sex or anthropometry required medical students and surgical residents to perform simulated tasks. Therefore, it is difficult to make inferences about the performance of more experienced laparoscopic surgeons in an operating environment. Secondly, reporting bias within the included studies may mean that articles were more likely to only report significant results, especially for secondary outcomes. This may increase the disparity of results between biological sexes or those of different glove sizes. The decision to exclude studies that only gave a level of statistical significance without corresponding numerical results would have also impacted the review. However, studies excluded for this reason included those showing both significant and non-significant differences based on demographic factors. Thirdly, there is a possibility that some imprecision was introduced during the literature synthesis. Some studies reported results graphically. In these instances, WebPlotDigitizer was used to obtain a numerical result from pixel locations on the graph. The accuracy of this process is limited by graph quality. Additionally, there were instances where there were unexplained inconsistencies in the presented data. Where possible, the authors were contacted to provide clarification. Otherwise, inferences were made, or the results were omitted.

Almost half of the meta-analyses produced an I^2^ value greater than 50%, a possible indicator of moderate to high heterogeneity. However, this should be interpreted cautiously. Efforts were made to only combine data of the same type (completion times of standardized tasks, injury prevalence, height of surgeons reporting pain, and grip styles). The I^2^ value suggests high variability in the completion times of the intracorporeal suturing, rope pass, and paper cut tasks; however, individual studies all consistently show that female surgeons took longer to finish these exercises. Regarding pain prevalence, the two anatomic regions associated with a significantly higher number of complaints among female surgeons, the neck and shoulders, showed I^2^ values below 25%. These levels of heterogeneity are acceptably low. The variability observed in the other anatomic regions could be related to demographic factors, sampling, or a smaller proportion of surgeons experiencing discomfort in these regions (potentially creating larger relative differences between males and females). Five of the six meta-analyses for grip styles were homogeneous, although increased bias can occur in meta-analyses of such a small number of studies. Overall, given the considerations discussed above, it is considered reasonable to place confidence in the results where significant differences were found based on biological sex.

In conclusion, there is some suggestion in the collated data that laparoscopic tools are not suitably designed for female and small-handed surgeons, which may contribute to longer completion times of standardized tasks, increased neck and shoulder pain, and self-reported difficulty with various instruments. Poor tool design would contribute to lower intraoperative comfort and performance, as would demographic factors. These findings regarding the importance of tool fit are also applicable to robotic console design. No consistent trend was found regarding surgeon height and physical discomfort. Additionally, no evidence was found regarding what bearing these results have on operating room performance. Research examining the impact of poor tool fit on the surgical performance of experienced surgeons based on biological sex and glove size would be beneficial for this area of investigation. Possibilities for creating highly customizable or adjustable tool handles also warrant further exploration.
